# Index-Catalogue of the Library of the Surgeon-General's Office, United States Army

**Published:** 1892-06

**Authors:** 


*Index-Catalogue of the Library of the Surgeon-General's Office, United States Army.* Vol. XII. REGER—SHUTTLEWORTH. Washington: GOVERNMENT PRINTING OFFICE. 1891.

We gladly welcome another volume of this gigantic undertaking, for which the profession owes everlasting gratitude to Dr. Billings and his helpers, who have been enabled, through the munificence of the United States Government, to do such vast and useful work. The more we become acquainted with it the more thoroughly we realise how indispensable it is.

